# Multiprofessional Interventions for Frailty in Patients with Heart Failure: A Comprehensive Review

**DOI:** 10.1007/s11897-025-00727-8

**Published:** 2025-11-08

**Authors:** Izabella Uchmanowicz, Maria Jędrzejczyk, Christopher S. Lee, Loreena Hill, Cristiana Vitale, Quin E. Denfeld, Ercole Vellone, Bernadetta Żółkowska, Sara Janczak, Asriel Juvenal Chamos, Marta Kałużna-Oleksy, Marta Wleklik, Magdalena Lisiak, Katarzyna Lomper

**Affiliations:** 1 Division of Research Methodology, Department of Nursing, Faculty of Nursing and Midwifery, Wroclaw Medical University, Wrocław, Poland; 2 Centre for Cardiovascular Health, Edinburgh Napier University, Sighthill Campus, Edinburgh, UK; 3 Boston College William F. Connell School of Nursing, Chestnut Hill, MA USA; 4 Department of Emergency Medical Service, Faculty of Nursing and Midwifery, Wroclaw Medical University, Wroclaw, Poland; 5 School of Nursing and Paramedic Science, Ulster University, Londonderry, UK; 6 San Raffaele Open University of Rome, Rome, Italy; 7 IRCCS San Raffaele, Rome, Italy; 8 School of Nursing, Oregon Health & Science University, Portland, OR USA; 9 Department of Biomedicine and Prevention, University of Roma Tor, Vergata, Italy; 10 Student Research Club of Heart Diseases, Faculty of Medicine, Wroclaw Medical University, Wrocław, Poland; 11 General and Transplant Surgery, Students’ Scientific Club of Vascular, Wroclaw Medical University, Wroclaw, Poland; 12 1st Department of Cardiology, University of Medical Sciences, Poznan, Poland; 13 Institute of Heart Diseases, University Hospital, Wroclaw, Poland; 14 University Hospital, Wroclaw, Poland; 15 School of Health & Social Care, Edinburgh Napier University, Edinburgh, UK

**Keywords:** Frailty, Heart failure, Multiprofessional care, Physical rehabilitation, Nutritional support

## Abstract

**Purpose of Review:**

Frailty is a prevalent and clinically significant condition affecting up to 45% of adults with heart failure (HF). Frailty reflects a state of reduced physiologic reserve and vulnerability to stressors, which profoundly influences health outcomes including health care utilization and survival. Frailty and HF interact through multiple overlapping pathophysiologic mechanisms, including chronic inflammation, neurohormonal abnormalities, and multiorgan dysfunction. This synergy, for the patient leads to poor physical performance, reduced independence, and a greater susceptibility to complications.

**Recent Findings:**

Evidence from recent clinical trials focused on frailty in HF underscores the potential benefits of multicomponent interventions combined with medication optimisation target physical dysfunction, poor nutrition, psychological disorders, and social isolation. Multidisciplinary care teams – including cardiologists, nurses, physical therapists, dieticians, occupational therapists, and social workers – can implement tailored strategies to reverse or slow the progression of frailty to improve health outcomes of patients with HF. Interventions such as resistance and balance training, individualised nutritional supplementation, medication review, cognitive and psychological support, and caregiver education have demonstrated a range of benefits in HF, from enhanced physical capacity and reduced hospital readmissions to improved health-related quality of life.

**Summary:**

Managing frailty in patients with HF requires a personalised, holistic and multicomponent approach. Successful intervention involves addressing not only physical dysfunction but also psychological, nutritional, and social factors that collectively undermine health and independence. Integrating multicomponent care into routine practice has the potential to improve clinical outcomes, reduce health care utilisation, and enhance the overall well-being of patients. Future research should aim to identify the most effective combinations of interventions, clarify mechanisms of action, and determine cost-efficiency in health care delivery.

## Introduction

Frailty is common among patients with heart failure (HF), affecting up to 45% of those with chronic disease and even more with acute decompensation [1, 2]. Both conditions share overlapping mechanisms – chronic inflammation, neurohormonal and mitochondrial dysfunction, and multiorgan impairment – that diminish physiologic reserve and muscle mass [3–11].

Frailty in HF is consistently associated with poor outcomes, including increased mortality, hospitalisations, functional decline, and reduced quality of life [2–5, 12, 13]. Because frail patients have limited stress tolerance, they also account for disproportionate healthcare use [6]. The relationship between frailty and HF is bidirectional, as each condition accelerates the progression of the other [7].

Mechanistic pathways such as inflammation, energy dysregulation, and neurohormonal imbalance contribute to weakness and disability [8–11]. Clinically, frailty can obscure or mimic HF symptoms, complicating diagnosis and treatment [14]. Yet frailty may be reversible when identified early and managed appropriately [15]. Incorporating frailty screening into routine HF care enables tailored interventions for those most at risk [16].

A multiprofessional model – integrating cardiologists, nurses, physiotherapists, dietitians, occupational therapists, and social workers – offers the most comprehensive approach [17–20]. Such collaborative care can reduce complications and readmissions while improving function and quality of life [21–27]. This review summarises and critically appraises evidence on multiprofessional interventions that target physical, nutritional, psychological, and social dimensions of frailty in patients with HF.

## Methods

This review was designed to critically synthesise recent evidence on multiprofessional interventions for frailty in patients with HF. The aim was to include high-quality original studies, clinical trials, systematic reviews, and practice guidelines that reflect international current multidisciplinary clinical practice.

### Search Strategy and Study Selection

A structured literature search was conducted in PubMed, Embase, and the Cochrane Library to identify relevant articles published between January 2015 and April 2025. Eligible studies included original research (randomised controlled trials, cohort and case-control studies), systematic reviews, meta-analyses, and clinical practice guidelines. Articles not published in English were excluded. Search terms were applied using both MeSH and free-text keywords, including: “frailty”, “heart failure”, “cardiac failure”, “multiprofessional”, “multidisciplinary”, “interdisciplinary”, “multidomain rehabilitation”, “integrated care”, “comprehensive intervention”, and specific components such as “physiotherapy”, “occupational therapy”, “nutrition”, “dietetic”, “psychology”, “social work”, and “nursing interventions.”

Titles and abstracts were screened for relevance, followed by full-text assessment against predefined inclusion criteria. Studies were included if they focused on adults with HF and frailty and described multicomponent or interdisciplinary interventions addressing two or more care domains. Discrepancies in study selection were resolved through discussion and consensus among reviewers. Only studies deemed relevant, methodologically sound, and sufficiently detailed in their description of interventions were included in the synthesis.

## Results

### Physiotherapy and Occupational Therapy

Physical dysfunction is a major challenge for patients with frailty and heart failure (HF), limiting independence, daily activities, and quality of life [28, 29]. Evidence, particularly from the REHAB-HF trial, highlights the benefits of multicomponent physical rehabilitation that includes aerobic, resistance, balance, and mobility training tailored to individual health status and goals [30, 31]. This approach significantly improves physical function, with the greatest benefits seen in the most frail patients. Occupational therapy complements physiotherapy by supporting activities of daily living (ADLs), adapting home environments to reduce falls, and addressing cognitive barriers to independence [28]. Together, these therapies enhance functional capacity, promote independence, and improve quality of life in frail HF patients.

### Nutritional Support and Dietetics

Malnutrition is common in frail patients with HF and is associated with worse outcomes, including increased mortality [32, 33]. Contributing factors include poor appetite, medication effects, fatigue, and limited food preparation ability [34]. Early detection using tools such as the Mini Nutritional Assessment (MNA) is essential [35, 36]. Individualised nutrition plans with adequate protein intake (1–1.5 g/kg/day) help maintain muscle mass and function [34, 37–39]. Correcting deficiencies in micronutrients like zinc, vitamin D, and iron can improve outcomes [40–46]. Sodium, fluid, potassium, and magnesium should be carefully managed to prevent complications [47–50]. Omega-3 fatty acids may provide additional benefit in selected cases [51]. A personalised, comprehensive nutritional approach is essential in managing frailty in HF [52–55].

### Psychological and Social Interventions

Psychosocial factors such as depression, cognitive decline, and social isolation significantly influence outcomes and treatment adherence in frail HF patients [56]. Lower education, poor social support, and reduced community participation contribute to frailty progression [57–62]. Multidimensional interventions targeting these areas can slow frailty and improve health outcomes [63, 64]. The SUNFRAIL + tool assesses physical, psychological, and social aspects of frailty to guide individualized care [63].

Higher education and cognitively demanding occupations may offer protection, with each year of education reducing frailty risk by about 2.6% [60]. Active social participation, including frequent contact with relatives and friends, is linked to lower frailty prevalence [65, 66]. Nurse-led, multicomponent interventions – combining cognitive training, education, and lifestyle changes – improve engagement and outcomes [67].

Addressing depression and sleep disorders is also essential [62]. Interventions such as cognitive behavioral therapy, social support groups, and caregiver education reduce psychological stress, enhance independence, and support treatment adherence. Together, these strategies highlight the importance of integrating psychological and social care into a comprehensive, patient-centered approach.

### Core Components of Multicomponent Interventions

Our review identified various strategies targeting frailty in heart failure patients. As frailty and HF often coexist and worsen outcomes, care should be individualized based on whether the main goal is managing frailty in HF or optimizing HF treatment in frail patients. Differentiating between cardiovascular (CV) and non-cardiovascular (non-CV) causes of frailty may further refine care approaches.

Multicomponent, multiprofessional interventions consistently improve outcomes such as reducing hospitalizations, enhancing function, and lowering mortality risk [7]. Key components, recommendations, and references are summarized in Table 1.

**Table 1:** Key intervention areas in frailty management for patients with heart failure

Intervention area	Key elements	Recommendations	References
Pharmacotherapy	- Individualising drug regimens - Considering physiological reserve, potential side effects, and drug-drug interactions - Close monitoring of diuretics, beta-blockers, ACE inhibitors - Minimising polypharmacy - Regular medication reviews	- Avoid worsening weakness and cognitive dysfunction - Reduce risk of adverse effects - Improve treatment safety and effectiveness	[7, 68]
Nursing care and education	- Assessment of symptoms, medication adherence, and nutritional status - Use of validated screening tools (e.g., MNA) to detect malnutrition early - Nurse-led education for patients and caregivers	- Early detection and intervention in malnutrition - Improved capacity of patients and caregivers to manage HF and frailty at home	[69, 70]
Physical activity and rehabilitation	Structured exercise programs: resistance, balance, and aerobic training - Tailoring interventions to patient preferences, goals, and comorbidities	- Improved physical capacity and quality of life - Reduced hospital readmissions - Even highly frail, multimorbid patients benefit (e.g., LIFE-P study)	[71, 72]
Psychosocial and cognitive support	- Nurse-led interventions: memory exercises, psychoeducation, cognitive training - Emotional and social support	- Reduced depression, anxiety, and social isolation - Improved patient engagement and mental well-being	[56, 67, 73]
Transitional care	- Comprehensive discharge planning - Telemonitoring and follow-up phone calls - Support for caregivers	- Reduced hospital readmissions - Improved continuity and safety of care	[74, 75]

In summary, the literature supports implementing multicomponent interventions addressing overlapping domains of frailty and heart failure – including medication optimization, physical rehabilitation, nutritional and psychosocial support, and education. Effective delivery requires coordinated multidisciplinary teams and tailoring to individual patient needs and capacities.

## Discussion

This review highlights the multidimensional character of frailty in HF, encompassing physical, cognitive, psychological, nutritional, and social domains. Addressing these interrelated aspects requires a multicomponent strategy that integrates interventions across disciplines to deliver coordinated and patient-centred care.

The strongest evidence supports tailored, multicomponent programmes targeting physical performance, nutrition, psychological well-being, and social support – mirroring the overlapping mechanisms of frailty and HF. Effective delivery depends on multidisciplinary collaboration among cardiologists, nurses, physiotherapists, dietitians, and social workers to ensure comprehensive support.

The REHAB-HF trial showed that multidimensional rehabilitation significantly improved physical function and quality of life, particularly in the most frail patients [30, 31]. Integrating nutritional therapy, medication optimisation, and psychosocial support enhances outcomes further. However, engaging frail individuals in sustained physical activity remains challenging; personalised goals, early positive feedback, and caregiver involvement can improve adherence [76].

Combining nutritional supplementation with resistance training may counteract sarcopenia, improve strength, and reduce readmissions [39]. Management of micronutrient deficiencies, sodium intake, and electrolyte balance should also be individualised [40–50]. Patient education and self-care are essential for maintaining long-term benefits.

Psychological and social dimensions are equally relevant. Depression, anxiety, and isolation frequently co-occur with frailty and worsen prognosis [51–56]. Interventions including counselling, peer support, and caregiver education can mitigate these effects and enhance treatment engagement.

Medication review and deprescribing constitute another key component of multiprofessional care. Optimising pharmacotherapy helps minimise polypharmacy-related adverse events, improve tolerance to guideline-directed therapies, and preserve functional capacity [57–63].

Although these interventions show promise, existing evidence is limited by heterogeneity in study design, frailty definitions, and intervention components. Most trials are small, short-term, and lack standardised outcome measures. Consequently, firm conclusions on the most effective models cannot yet be drawn. Larger, well-designed randomised studies using consistent frailty assessments and long-term follow-up are urgently needed.

### Limitations

This review is subject to several limitations. First, the search was limited to English-language publications, which may have introduced language bias. Second, heterogeneity in frailty definitions, assessment tools and intervention components precluded formal meta-analysis and reduced comparability across studies. Third, many included studies had small sample sizes and short follow-up, limiting inference about long-term effectiveness and generalisability. Finally, publication bias and selective reporting may have skewed the apparent benefits of interventions. These limitations should temper interpretation and highlight the need for standardized, larger, and longer trials.

### Clinical Implications

Implementing multicomponent interventions for frail patients with HF can translate directly into clinical practice by fostering holistic, patient-centered care. Routine screening for frailty should become an integral part of HF management, guiding individualized treatment plans that incorporate exercise, nutritional support, psychosocial counseling, and social engagement. Integration of these components within multidisciplinary care teams may reduce hospital readmissions, improve functional capacity, and enhance patients’ quality of life. Furthermore, embedding frailty management into existing HF care pathways can promote continuity of care and optimize resource utilization across healthcare settings.

### Future Perspectives

Future studies should aim to identify which combinations of intervention components yield the greatest benefits for specific subgroups of frail HF patients. Longitudinal trials are needed to evaluate the long-term sustainability, cost-effectiveness, and scalability of multicomponent models in real-world clinical settings. Moreover, research should explore the role of emerging technologies – such as telemonitoring, digital rehabilitation platforms, and artificial intelligence – assisted assessment in supporting personalized frailty management and early detection of functional decline.

## Conclusion

Multiprofessional, multicomponent interventions targeting physical function, nutrition, medication optimisation and psychosocial needs show potential to improve functional capacity, quality of life and reduce readmissions among frail patients with heart failure. However, evidence is heterogeneous and largely derived from small or short-term studies. Large-scale randomized trials with standardized frailty measures and cost-effectiveness analyses are needed to define optimal, scalable care models.

## Key References

- Kitzman DW, Whellan DJ, Duncan P, Pastva AM, Mentz RJ, Reeves GR, et al. Physical Rehabilitation for Older Patients Hospitalized for Heart Failure. N Engl J Med. 2021;385(3):203–216.
  - A landmark randomized trial (REHAB-HF) demonstrating that tailored, multidomain rehabilitation significantly improves physical function and quality of life in frail patients with heart failure.
- Pandey A, Kitzman DW, Reeves GR. Frailty Is Intertwined With Heart Failure: Mechanisms, Prevalence, Prognosis, Assessment, and Management. JACC Heart Fail. 2019;7(12):1001–1011.
  - This comprehensive review details shared pathophysiological mechanisms and underscores the importance of integrated frailty assessment in the management of heart failure.
- Vitale C, Jankowska EA, Hill L, et al. Heart Failure Association/European Society of Cardiology position paper on frailty in patients with heart failure. Eur J Heart Fail. 2019;21(11):1299–1306.
  - Presents expert consensus recommendations endorsing multiprofessional care models to meet the complex needs of frail patients with heart failure.
- Uchmanowicz I, Lisiak M, Lomper K, et al. State of the Art in Measuring Frailty in Patients With Heart Failure: from Diagnosis to Advanced Heart Failure. Curr Heart Fail Rep. 2025;22(1):11–22.
  - Highlights validated assessment tools to identify physical, psychological, and social frailty domains in heart failure populations, supporting early identification and stratification.
- De Luca V, Donnoli C, Formosa V, et al. Preliminary results of a multidimensional approach to screen for frailty in community-dwelling older adults: the SUNFRAIL + study. Front Public Health. 2025;13.
  - Demonstrates that biopsychosocial frailty screening tools enhance early detection and guide the planning of individualized, multiprofessional interventions.

## Data Availability

No datasets were generated or analysed during the current study.
